# Uncovering the gaps: a grounded theory approach to conceptualizing inadequate child family caregiving in China

**DOI:** 10.3389/fpubh.2025.1539227

**Published:** 2025-08-06

**Authors:** Zhang Jia-Yuan, Yang Jinwei, Zhou Yuqiu

**Affiliations:** ^1^Department of Psychological Nursing, Harbin Medical University, Daqing, China; ^2^Department of Medicine, Huzhou University, Huzhou, China

**Keywords:** children, family care, grounded theory, qualitative, conceptual framework

## Abstract

**Objective:**

This study investigates the manifestations of inadequate family caregiving for children in China and aims to construct a conceptual framework that captures the multifaceted nature of this phenomenon within a rapidly changing social context.

**Methods:**

Based on constructivist grounded theory, in-depth interviews were conducted with 28 children and their primary family caregivers across diverse socioeconomic backgrounds, selected through purposive and theoretical sampling. Through open, axial, and selective coding, this study identified and refined the core attributes, antecedents, and outcomes of inadequate family caregiving, ultimately developing a conceptual framework.

**Results:**

Through the coding process, four core dimensions of caregiving insufficiencies were identified: daily living care, emotional and psychological support, safety supervision, and educational guidance. These gaps in caregiving were found to stem from a combination of internal family factors, such as unstable caregiving structures, limited parenting knowledge, low motivation, and insufficient caregiving skills, as well as external challenges, including limited family and community support and sociocultural pressures. The study found that these caregiving gaps were linked to strained parent–child relationships, delays in social and emotional development, behavioral issues, and potential risks to children’s physical health.

**Conclusion:**

Inadequate family caregiving emerges from a mix of internal and external constraints that limit caregiving capacities and resource access, resulting in multidimensional caregiving deficiencies that impact children’s physical and mental health. Addressing this issue necessitates enhancing family caregiving capacities, strengthening social support networks.

## 1 Introduction

The family environment forms the primary sphere in which a child grows, significantly impacting the child’s physical, psychological, and social development (1). Effective family functioning is vital for holistic child development, with caregiving as a pivotal mechanism for fulfilling this role. Quality caregiving within the family meets children’s diverse needs and provides critical support for their health and comprehensive growth (2). Studies have revealed a strong correlation between family caregiving quality and children’s mental and physical well-being. An increasing body of research links prevalent issues—such as anxiety, depression, behavioral problems, learning difficulties, and social challenges—with suboptimal family interactions, inadequate caregiving, and lack of emotional support (3, 4). Positive family caregiving is essential for fostering children’s emotional health, social skills, and academic success, whereas caregiving deficiencies can worsen adverse mental and physical developmental outcomes (5).

In recent years, children’s physical and mental health needs have become increasingly complex, particularly in societies undergoing rapid transformation, such as China (6). Academic competition, digital media exposure, family separation, and shifting social expectations have intensified the pressure on both children and caregivers (6). National surveys have shown a rising prevalence of emotional and behavioral problems among Chinese children and adolescents, emphasizing the pressing need to understand the gaps in caregiving systems’ responses to these changing demands (7, 8).

Against this backdrop, the concept of inadequate family caregiving has gained increasing relevance. Yet, despite its importance, the notion remains inconsistently defined and under-theorized, particularly in non-Western contexts. Existing studies overlook more common but persistent forms of caregiving deficits, such as lack of emotional availability, poor supervision, or inconsistent educational support (9, 10). In China, as socioeconomic conditions evolve and urbanization accelerates, family structures and lifestyles are undergoing substantial changes, creating complex challenges in child caregiving (11).

In recent years, rapid internal migration and sweeping social changes have led to increasingly fragmented and unstable caregiving arrangements across diverse family contexts in China. This fragmentation is driven by structural factors such as urban–rural inequality, household registration barriers, and shifting labor demands (12). First, many urban migrant families live with their children but face long working hours and limited access to public services, leaving their children with insufficient emotional and educational support (13). Second, although the number of “left-behind children”—those remaining in rural areas while their parents work in cities—has declined, many still lack consistent supervision and psychological care because of persistent regional income disparities (14). Third, even in urban middle-class households, the rise of dual-income families has led to time poverty and weakened intergenerational support, undermining parents’ caregiving capacity (15). Compounding these structural constraints are changing family values that prioritize academic performance and individual success, often at the cost of emotional connections. These overlapping social, economic, and cultural pressures reveal not only how caregiving is strained but also why existing definitions of inadequate caregiving may be insufficient to capture the full complexity of everyday caregiving challenges in contemporary China (16).

To address this gap, this study adopts grounded theory as a methodological approach to develop a systematic conceptual framework for inadequate caregiving in China. Grounded theory is a qualitative methodology designed to build theories through iterative data collection, coding, and theoretical abstraction. It enables researchers to identify core concepts that reflect social phenomena and map the relationships among these concepts to form cohesive theoretical models (17). The current literature on inadequate caregiving remains limited and fragmented, with few robust theoretical models, particularly in non-Western contexts, posing challenges for quantitative analysis and cross-cultural comparison. In addition, caregivers from diverse backgrounds often hold vague or inconsistent views on what constitutes inadequate caregiving, which reduces the effectiveness of structured questionnaires in large-sample studies. By generating theory directly from empirical data, this study aims to extract the key dimensions and meanings of inadequate caregiving as experienced by contemporary Chinese families. The resulting framework is intended to guide future intervention design, policy development, and culturally grounded caregiving research, ultimately supporting healthier developmental outcomes for children in the future.

## 2 Methods

### 2.1 Participants

To capture diverse caregiving experiences, we employed purposive and theoretical sampling to recruit children and their primary caregivers from both urban and rural areas in four provinces—Heilongjiang, Guangdong, Guizhou, and Hubei—selected for their variations in economic development, cultural background, and geographic context. Recruitment was facilitated through local schools, community health centers, and neighborhood committees from January to March 2024. No incentives were provided for participation. Prior to enrollment, caregivers completed brief pre-screening questions to ensure adequate communication abilities during the interview process. Children with acute or chronic illnesses were excluded to maintain a focus on general family caregiving rather than care shaped by specific medical needs. The inclusion criteria for family caregivers were as follows:

① Aged 18 years or older.
② Served as the primary caregivers of the child.
③ Capable of clear communication.
④ Willing to participate after understanding the study objectives.

The children included in the study were aged 8 to 16 years, able to express their thoughts and feelings, and participated with guardian consent and personal willingness.

The exclusion criteria were as follows:

① Non-family caregivers (such as nannies or professional caregivers).
② Caregivers with mental health or cognitive impairments that might compromise response accuracy.
③ Children with acute or chronic illnesses.

The sample size was determined based on the principle of data saturation at which point theoretical saturation was confirmed. Therefore, the final sample included 28 children and their primary caregivers. Detailed participant information is provided in Table 1.

**Table 1:** The details information of participants.

No.	Province	Residence	Age of child	Gender of child	Relations with child	Age of caregiver	Socioeconomic status	Family type*
01	Heilongjiang	Urban	10	Boy	Mother	38	Middle-income	Core family
02	Heilongjiang	Rural	8	Girl	Grandmother	65	Low-income	Left-behind
03	Guangdong	Urban	12	Boy	Father	42	High-income	Core family
04	Guangdong	Rural	9	Girl	Mother	35	Low-income	Core family
05	Guizhou	Urban	11	Girl	Mother	40	Middle-income	Single-parent family
06	Guizhou	Rural	9	Boy	Mother	36	Low-income	Immigrant
07	Hubei	Urban	14	Boy	Mother	39	Middle-income	Core family
08	Hubei	Rural	16	Girl	Mother	41	Low-income	Left-behind
09	Heilongjiang	Urban	13	Girl	Mother	40	High-income	Core family
10	Guangdong	Urban	15	Boy	Mother	37	Middle-income	Immigrant
11	Guizhou	Rural	10	Girl	Mother	43	Low-income	Core family
12	Hubei	Rural	9	Girl	Mother	35	Middle-income	Immigrant
13	Guangdong	Rural	11	Boy	Grandmother	64	Low-income	Left-behind
14	Heilongjiang	Urban	12	Girl	Mother	41	Middle-income	Immigrant
15	Guizhou	Urban	15	Boy	Mother	36	Low-income	Core family
16	Hubei	Rural	10	Girl	Grandfather	67	Low-income	Left-behind
17	Guangdong	Urban	13	Boy	Father	44	High-income	Core family
18	Heilongjiang	Rural	14	Girl	Grandmother	62	Low-income	Left-behind
19	Hubei	Urban	16	Boy	Mother	38	Middle-income	Core family
20	Guizhou	Rural	12	Boy	Mother	34	Low-income	Core family
21	Guangdong	Urban	11	Boy	Mother	39	High-income	Single-parent family
22	Heilongjiang	Urban	13	Girl	Mother	37	Middle-income	Core family
23	Hubei	Rural	9	Boy	Grandmother	66	Low-income	Left-behind
24	Guizhou	Urban	12	Girl	Mother	42	Low-income	Single-parent family
25	Guangdong	Rural	13	Boy	Grandmother	70	Low-income	Immigrant
26	Hubei	Urban	10	Girl	Mother	36	Middle-income	Core family
27	Heilongjiang	Rural	14	Boy	Mother	35	Middle-income	Immigrant
28	Guizhou	Rural	16	Boy	Mother	40	Low-income	Core family

*Family type: Core family:Nuclear families consisting of parents and their children living together in the same correct household; Core family: Nuclear families consisting of parents and their children living together in the same household; Left-behind families: Families where one or both parents have migrated to urban areas for work, leaving children and/or older adult in the rural home area; Single-parent families: Families headed by one parent, either due to divorce, separation, death of spouse; Immigrant families: Families who migrated from rural areas to urban areas within China.

### 2.2 Ethics statement

The study protocol was reviewed and approved by the Ethics Committee of Harbin Medical University, Daqing Campus (No. HMUDQ20231212009), ensuring compliance with ethical standards for research involving human participants. Informed consent was obtained from all the participants.

### 2.3 Data collection

To delve into the phenomenon of inadequate family caregiving for children, a semi-structured interview approach was used. The interview guide was initially created by reviewing the relevant literature and materials. After a pilot study and feedback from experts, the guide was refined and finalized (see Table 2). Prior to the interviews, the research team underwent specialized training to develop key interview techniques, including active listening, clarification and probing. After a preliminary round of interviews, minor adjustments were made to the questions and topics, based on participant feedback, to better capture pertinent information. Formal interviews were conducted from January to March 2024, comprising face-to-face, in-depth discussions with each participant. Each session lasted approximately 30–60 min. Before starting, the participants were briefed on the study’s purpose and methods, and consent was obtained to record the conversations. Following each interview, a summary report was drafted to document the key insights. All recordings were transcribed within 24 h and reviewed for accuracy. After obtaining confirmation from the participants, 56 complete transcripts were finalized, consisting of 28 interviews with children and 28 with caregivers.

**Table 2:** The interview outline.

Subject	Questions
Children	Who primarily takes care of you? Do you think they spend enough time taking care of you?
	When you need help, do family members respond promptly? If not, how do you handle it?
	In what areas do you feel well cared for at home? Are there areas where you feel care might be lacking?
	Have you ever felt that family members were not taking care of or showing concern for you? Could you give examples?
	At school or when chatting with friends, do you feel any differences in family care compared to others? Could you give examples?
	What changes would you like to see in the way your family cares for you?
	How do you think your current family caregiving situation affects you? For example, in your studies, emotions, or interactions with friends?
	If you had the chance to express your caregiving needs to your parents or family, what would you say?
Family caregiver	What aspects of child caregiving are you primarily responsible for in the family?
	Could you describe the specific details of how you care for your child?
	Do you feel there are any areas where caregiving may be insufficient?
	What areas does this primarily include?
	Has your child ever expressed feelings about family care and suggested changes? What specific areas were mentioned?
	In your opinion, what factors might contribute to insufficient care for your child?
	Do you think your child’s behavior is influenced by a lack of family care? Have you observed any noticeable changes in them?

### 2.4 Data analysis

This study utilized a three-phase coding method based on grounded theory to analyze the phenomenon of inadequate family caregiving for children: ① Open Coding: Interview data from children and parents were deconstructed, identifying key concepts and themes related to caregiving deficiencies. ② Axial Coding: Feedback from different respondents was integrated, allowing for a comparison of commonalities and differences concerning care deficiencies. This phase helped identify the main categories and their relationships. ③ Selective Coding: Core concepts were extracted and linked with their antecedents and consequences, forming the basis for a conceptual framework. This process facilitated the construction of a comprehensive, conceptual framework. Throughout the analysis, emphasis was placed on integrating both children’s experiences and parents’ perspectives to ensure that the resulting theoretical model reflected the multifaceted impacts of caregiving deficiencies. The final conceptual framework provides a detailed understanding of the core attributes, underlying causes, and significant impacts of inadequate family care on child development, highlighting the complex contexts and resulting effects on children’s growth.

## 3 Results

### 3.1 General information of participants

The study included 28 children and their family caregivers as interview participants, with an equal distribution of 14 each from urban and rural areas. The caregivers were composed of 20 mothers, 2 fathers, and 6 grandparents. Regarding the children, 15 were boys and 13 were girls. The average age of the children was 12.04 years (SD = 1.38), with ages ranging from 8 to 16 years old. Family structure data indicated that 13 families had only one child and 15 families had more than one child. This diverse sample covered a variety of genders, family roles, urban–rural backgrounds, and family structures, providing a comprehensive perspective for analyzing inadequate child family caregiving.

### 3.2 Open coding

Open coding is a foundational step in qualitative analysis that involves conceptualizing raw data line by line and sentence by sentence. This study employed a combination of line-by-line, sentence-by-sentence, and paragraph-level coding techniques to ensure that all concepts, including subtle ones, were identified comprehensively. During this phase, 436 initial concepts were extracted from interviews with children, and 631 from caregiver interviews. These concepts were refined and abstracted into higher-order categories suitable for theoretical analysis. By consolidating repetitive, related, or loosely structured concepts, the study generated 35 preliminary categories. Table 3 presents examples of initial concepts and their corresponding categories, demonstrating how raw data were transformed into meaningful analytical units.

**Table 3:** Preliminary categories.

Code	Initial category	Initial concept
N1	Nutritional Imbalance in Children’s Food	A2-3: Unbalanced diet structure, mainly meat-based with a lack of dietary fiber; A15-2: Over-reliance on high-protein foods (e.g., beef, eggs) with insufficient vegetable and grain intake; C5-2: Excessive intake of high-calorie foods (e.g., sweets, fried foods) with a lack of fruit and whole grains.
N2	Excessive Intake of Unhealthy Foods by Children	C4-5: Children frequently consume high-sugar, high-fat foods, such as drinks and chips; A7-8: Children regularly eat fried foods with minimal intake of vegetables and fruits; C9-10: Due to busy schedules, parents often buy fast food and convenience foods for children, neglecting nutritional balance.
N3	Poor Eating Habits in Children	A17-15: Children tend to be picky eaters and avoid nutritious foods; A22-17: Children watch TV or play on phones while eating, leading to a lack of focus during meals.
N4	Insufficient Sleep and Rest Time for Children	C26-21: Parents fail to manage routines effectively; children participate in excessive extracurricular activities, resulting in late nights, early mornings, and insufficient rest; C15-24: Children often stay up late due to phone or tablet use, leading to inadequate sleep.
N5	Irregular Sleep Patterns in Children	C17-23: Lack of parental guidance causes children to stay up on weekends playing games or watching TV, disrupting regular sleep routines; C23-19: Parents frequently stay up late and lack supervision, resulting in children developing irregular and inconsistent sleep schedules.
N6	Insufficient Physical Activity in Children	C6-31: Children lack outdoor activities; A8-24: Parents prioritize academic activities, preventing children from engaging in regular physical exercise.
N7	Lack of Regular Health Monitoring for Children	A20-36: Children miss regular check-ups or health assessments as parents do not actively arrange health screenings; C11-43: Parents have insufficient understanding of children’s health status, often overlooking potential health or developmental issues.
N8	Improper Handling of Children’s Health Symptoms	A8-57: Parents neglect mild symptoms or discomfort in children, delaying medical attention; A25-42: Parents self-diagnose and administer home remedies without consulting doctors, worsening children’s conditions; A21-50: Parents rely on folk remedies or online advice, using unverified treatments.
N9	Poor Personal Hygiene Habits in Children	C16-63: Children lack regular hand-washing habits due to insufficient parental guidance and supervision; A24-43: Children do not maintain a habit of brushing teeth twice daily, with inconsistent parental guidance; A1-73: Children resist bathing or delay it, and parents do not enforce consistent cleanliness.
N10	Untimely Household Cleanliness	C14-73: Cleaning is infrequent, with toys and clothes seldom cleaned; C2-80: Parents neglect indoor hygiene, with dust accumulating on floors and furniture.
N11	Limited Parent–Child Time	C2-73: Parents are frequently busy with work, often working overtime or traveling, resulting in limited time with children; C13-86: Communication and interaction time with children are minimal, with a lack of daily companionship.
N12	Lack of High-Quality Parent–Child Interaction	A10-96: Although parents are present, most of their time is spent on work tasks, with minimal effective interaction; A8-113: Even when home, parents are often busy with household chores, missing opportunities to engage with children.
N13	Limited Parental Emotional Expression	A18-119: Parents provide little emotional response when children encounter problems; C2-93: Parents lack positive emotional expression toward children in daily life, leaving children feeling unloved.
N14	Neglect of Children’s Psychological and Emotional Needs	A2-131: Parents rarely take the initiative to understand children’s psychological states or emotional changes; C2-126: Parents overlook children’s internal feelings, lacking empathy and emotional connection.
N15	Ignoring Children’s Expressions	C11-124: Parents often dominate conversations with their own views; C28-133: Parents lack patience in listening to children’s questions or emotional expressions; A16-149: Parents fail to delve into children’s true thoughts and feelings.
N16	Lack of Understanding of Children’s Developmental Processes	A19-163: Parents misinterpret children’s behaviors; A16-170: Parents have overly high expectations for children’s abilities and cognitive levels; A12-181: Parents only focus on the outcome when children make mistakes, without understanding the reasons behind them.
N17	Insufficient Psychological Support	C17-147: Parents fail to provide effective coping strategies when children face stress or challenges; C13-153: During psychological difficulties, there is a lack of effective advice, guidance, or strategies to help children cope.
N18	Insufficient Emotional Support	C13-169: Parents focus excessively on pointing out children’s errors or shortcomings, lacking recognition and encouragement for their efforts and progress; A13-191: When children feel sad or anxious, parents choose to avoid or ignore these emotions.
N19	Inadequate Safety Supervision	C7-182: Dangerous household items (e.g., medication, knives) are not properly stored; A14-204: Parents seldom monitor children’s outdoor activities, failing to ensure their safety while outside.
N20	Limited Content or Methods in Safety Education	C2-198: Children are unaware of potential threats like cyberbullying, inappropriate content, or privacy leaks; A5-213: Parents forbid risky activities without teaching children to learn self-protection and risk assessment in safe contexts.
N21	Lack of Behavior Habit Development Education	C8-216: Parents fail to monitor and correct children’s bad habits; A9-241: Parents do not help children develop positive behavioral habits.
N22	Misplaced Educational Focus	C11-259: Parents focus solely on academic performance, neglecting the development of learning interest and other talents; A2-277: Parents do not teach children positive ways to handle stress and challenges, lacking stress management education; C26-264: Parents restrict children’s participation in socialization activities.
N23	Inappropriate Parenting Methods	C10-278: Parents fail to model positive behavior for children; C5-256: When children make mistakes, parents only criticize rather than guide and educate.
	Unstable Family Care Structure	A20-301: Frequent changes in caregivers or living environments within the family; A22-313: Multiple family members share caregiving responsibilities without clear roles.
N25	Insufficient Parenting Knowledge	A8-338: Parents rely on traditional child-rearing practices passed down from previous generations, lacking awareness of modern, science-based parenting knowledge; A18-356: Caregivers are unaware of children’s psychological needs and physical characteristics at various developmental stages.
N26	Lack of Motivation in Parenting	A14-379: Parents show a weak sense of responsibility for raising children, with limited involvement in their development; A12-406: Parents lack interest in the parenting process, showing little initiative in understanding children’s developmental progress and needs; A10-413: Parents view parenting as an unavoidable burden, feeling helpless and frustrated; A6-420: Parents prioritize career development or personal goals over spending time and energy on childcare.
N27	Limited Parenting Skills/Capabilities	A7-439: Low household income affects living conditions, educational resources, and healthcare; A16-457: Low education level impacts the ability to make informed parenting decisions; A20-463: Communication is overly directive or critical, lacking skills for effective dialogue with children.
N28	Insufficient Emotional Support within the Family	A28-490: Parents or guardians lack emotional support or assistance from partners or other family members during parenting; A20-513: Parents disagree on parenting methods, or grandparents interfere, leading to unclear caregiving responsibilities and conflicts in parenting approaches.
N29	Weak Social Support System	A21-527: Schools or communities fail to provide family education courses, lacking professional parenting and educational support; A5-536: Parents lack access to mental health services and support.
N30	Societal and Cultural Pressure	A7-574: Parents’ anxiety over academic performance causes them to neglect children’s emotional needs and daily care; A3-595: Parents are overly focused on comparing their children with others, overlooking individuality and emotional support; A9-608: Societal expectations stereotype fathers, leading to limited emotional and daily involvement from fathers.
N31	Decline in Parent–Child Relationship Quality	A13-613: Daily communication between parents and children decreases, lacking deep conversations and emotional connection; C10-271: Children feel neglected, weakening emotional bonds and making the relationship superficial; A15-620: Insufficient parental supervision of children’s behavior leads to confusion in behavioral norms, causing conflicts between children and parents.
N32	Limited Social Development in Children	C28-302: Children show more withdrawal or passivity in social situations, or struggle to handle social conflicts; C23-329: Children feel apprehensive and insecure when facing challenges, lacking confidence.
N33	Increase in Behavioral Problems in Children	C4-348: Children seek attention or express dissatisfaction through rebellious or disruptive behaviors; C6-356: Unable to process complex emotions and external pressures, children may resort to self-harm as an expression of distress.
N34	Emotional Imbalance in Children	C9-372: Children struggle to express emotions, opting to suppress them to avoid emotional interactions; C11-395: Children have a negative self-image, feeling “unworthy” of love or care; C25-401: Excessive anxiety over future events or minor issues; C20-415: Persistent self-doubt and low self-esteem regarding abilities and achievements.
N35	Health Issues in Children	A20-629: Lack of healthy lifestyle and adequate care leads to malnutrition or obesity; C2-426: Emotional issues and anxiety disrupt children’s ability to sleep or maintain healthy sleep habits.

### 3.3 Axial coding

Axial coding builds upon open coding by rearranging and recombining initial categories to uncover key categories that represent relationships among them. From the open coding results, this study identified 11 subcategories and 7 main categories through axial coding:

① Inadequate care in children’s daily lives.
② Inadequate care in children’s emotional and psychological well-being.
③ Inadequate care in children’s safety.
④ Inadequate care in children’s educational development.
⑤ Internal factors contributing to the lack of family care for children.
⑥ External factors contributing to the lack of family care for children.
⑦ Negative consequences of inadequate family care for children.

Table 4 presents each main category along with their corresponding subcategories, showcasing the comprehensive structure of identified categories.

**Table 4:** The main category and its corresponding subcategory.

Main category	Subcategory	Initial category	Code
Inadequate in daily living care	Inadequate Nutritional Care	Nutritional Imbalance in Children’s Food	A2-3; A15-2; C5-2
		Excessive Intake of Unhealthy	C4-5; A7-8
		Foods by Children	C9-10
		Poor Eating Habits in Children	A17-15; A22-17
	Insufficient Rest and Physical Activity Care	Insufficient Sleep and Rest Time for Children	C26-21; C15-24
		Irregular Sleep Patterns in Children	C17-23; C23-19
		Insufficient Physical Activity in Children	C6-31; A8-24
	Lack of Health Monitoring and Medical Care	Lack of Regular Health Monitoring for Children	A20-36; C11-43
		Improper Handling of Children’s Health Symptoms	A8-57; A25-42; A21-50
	Inadequate Hygiene and Cleanliness Care	Poor Personal Hygiene Habits in Children	C16-63; A24-43; A1-73
		Untimely Household Cleanliness	C14-73; C2-80
Inadequate in emotional and psychological care	Lack of Companionship	Limited Parent–Child Time	C2-73; C13-86
		Lack of High-Quality Parent–Child Interaction	A10-96; A8-113
	Insufficient Affection	Limited Parental Emotional Expression	A18-119; C2-93
		Neglect of Children’s Psychological and Emotional Needs	A2-131; C2-126
	Lack of Understanding	Ignoring Children’s Expressions	C11-124; C28-133
		Lack of Understanding of Children’s Developmental Processes	A19-163; A16-170; A12-181
	Insufficient Support	Insufficient Psychological Support	C17-147; C13-153
		Insufficient Emotional Support	C13-169; A13-191
Inadequate in safety care	—	Inadequate Safety Supervision	C7-182; A14-204
	—	Limited Content or Methods in Safety Education	C2-198; A5-213
Inadequate in educational care	—	Lack of Behavior Habit Development Education	C8-216; A9-241
	—	Misplaced Educational Focus	C11-259; A2-277; C26-264
	—	Inappropriate Parenting Methods	C10-278; C5-256
Internal factors contributing to inadequate family care for children	—	Unstable Family Care Structure	A20-301; A22-313
	Caregiver-Related Factors	Insufficient Parenting Knowledge	A8-338; A18-356
		Lack of Motivation in Parenting	A14-379; A12-406; A10-413
		Limited Parenting Skills/Capabilities	A7-439; A16-457
External factors contributing to inadequate family care for children	—	Insufficient Emotional Support within the Family	A28-490; A20-513
	Social Factors	Weak Social Support System	A21-527; A5-536
		Societal and Cultural Pressure	A7-574; A3-595
Adverse outcomes of inadequate family care for children	—	Decline in Parent–Child Relationship Quality	A13-613; C10-271; A15-620
	Physical and Mental Developmental Issues in Children	Limited Social Development in Children	C28-302; C23-329
		Increase in Behavioral Problems in Children	C4-348; C6-356
		Emotional Imbalance in Children	C9-372; C11-395; C25-401
		Health Issues in Children	A20-629; C2-426

### 3.4 Selective coding

**Figure 1:**
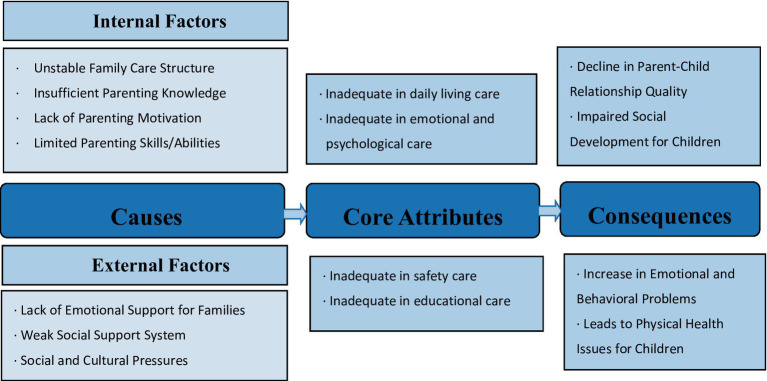
Conceptual framework of inadequate child family caregiving.

Selective coding focuses on identifying a core category and exploring its logical relationships with the main categories. In this study, a detailed analysis of 35 subcategories and 7 main categories led to the abstraction of a single core concept. This concept served as the foundation for constructing a conceptual framework for inadequate family care, encapsulating its core attributes, antecedents, and consequences. Figure 1 illustrates the conceptual framework developed through this analysis.

### 3.5 Theoretical saturation verification

The analysis confirmed that the category system was theoretically saturated, as no new categories emerged during the final stages. To validate this, four additional interviewees were included beyond the initial sample. However, no new concepts or categories were identified, confirming that theoretical saturation had been achieved in this study.

## 4 Discussion

This study applied grounded theory to analyze textual data on children’s family care, progressing through a three-phase coding process to identify 35 initial categories, 11 subcategories, and seven main categories, ultimately resulting in a conceptual framework for inadequate family care for children. This framework highlights the core attributes of inadequate family care, along with its antecedents and consequences, providing a comprehensive view of how caregiving deficiencies affect child development.

### 4.1 Core attributes of inadequate family care for children

The study identifies four core attributes of inadequate family care for children: insufficient daily living care, inadequate emotional and psychological support, lack of safety care, and poor educational care. These attributes significantly influence various developmental aspects and are closely linked to children’s fundamental growth needs (18). According to Maslow’s hierarchy of needs, meeting physiological needs is essential for child development, as it establishes a foundation upon which higher developmental needs are built (19). Basic components, such as proper nutrition, adequate sleep, and healthy habits, are vital for children’s overall well-being (20). However, the study reveals that many families face challenges in providing adequate daily living care, particularly concerning diet, sleep, and health monitoring. Common dietary issues include imbalanced meals and excessive intake of unhealthy foods, reflecting family factors that impact children’s eating behaviors (21). Participant quotes highlight these issues: “My son eats instant noodles almost every night because I get home too late to cook.” Another noted, “We know vegetables are good, but they complain and we just give up.” Furthermore, sleep insufficiency is widespread, with studies indicating that Chinese adolescents aged 6–17 sleep an average of 7.9 h, falling short of the recommended 8.5 h (22). One child reported, “I usually go to bed at midnight because I have to do the homework.” Irregular sleep patterns among children, often linked to family environments where parents undervalue the importance of sleep, exacerbate this issue. As another child put it, “Both my parents stay up very late, so I naturally developed the habit too.” Additionally, some families neglect regular health monitoring and medical care, leading to delayed intervention for health issues. One caregiver noted, “We never did routine checkups; it’s not necessary.” Regular health monitoring is crucial for disease prevention and early detection. However, less than 30% of families in China conduct health assessments for their children, highlighting a gap in preventive care (23).

Beyond fulfilling material needs, children require emotional support and psychological care within their family environment (24). However, both parents and children frequently mentioned emotional neglect and limited companionship, suggesting a shared recognition of caregiving gaps. While children tended to express feelings of being emotionally overlooked or unheard, parents often described uncertainty or helplessness in responding to their children’s emotional needs. One child remarked, “Mom is home, but she’s always on the phone.” These statements reflect how emotional neglect can persist even in physically intact family units. Research indicates that children deprived of parental companionship often experience loneliness and neglect and struggle to feel a sense of belonging and emotional security (25). Additionally, many parents have limited understanding of their children’s emotional needs. A parent said, “I just do not know what to say when she’s sad, so I stay silent.” This lack of understanding can hinder effective communication, prevent positive emotional feedback, and ultimately harm children’s mental health (26). In some cases, parents, although physically present, fail to provide adequate emotional support, leading to feelings of insecurity and increasing the risk of anxiety or depression (27). The family environment is critical for mental health, and studies have shown that positive parental support enhances children’s ability to cope with psychological challenges (28). Conversely, a lack of emotional support can result in unmet emotional needs, difficulties in emotional expression, and psychological stress, which can impact children’s social skills and their self-perception (29).

In the area of safety care, children’s limited awareness and self-protection abilities make them particularly vulnerable to various risks, underscoring the importance of consistent parental supervision (30). Inadequate home safety measures, such as unsecured furniture or lack of supervision during high-risk activities, further exacerbate these dangers (31). Deficiencies in safety education are evident in terms of content and delivery. Many parents restrict safety education to physical or traditional threats (e.g., “do not run with scissors”), while overlooking modern risks related to digital environments, such as cyberbullying, online predators, and privacy breaches. As one child shared, “My dad says not to talk to strangers, but I use the internet alone all the time.” This gap reflects a lack of digital literacy among caregivers, who may not fully grasp the scope of the contemporary threats children face. Moreover, some parents adopt overly simplistic or authoritarian methods to deliver safety messages, often relying on commands without explanations. One child reported, “My parents just tell me things like, ‘Do not ask why—just do what I say,’ without giving any explanation.” Such approaches may fail to engage children’s understanding or cooperation, thereby hindering the internalization of safety principles and the development of long-term awareness and risk-management skills. These findings highlight the need for more age-appropriate, dialogical, and up-to-date safety education practices in the family setting.

Family education involves holistic guidance provided by parents or family members through actions and words in their daily lives. It encompasses academic knowledge, moral values, behavioral habits, life skills, emotional regulation, and personal values (32). However, the study highlights several deficiencies in educational care. Parents frequently prioritize academic success over other things. One parent stated, “I just want her to get into a good school. Other things can wait.” Another reflected, “As long as his grades are okay, I do not care about anything else.” These attitudes may heighten psychological stress and narrow the children’s self-perception. Studies have shown that achievement-oriented parenting is associated with increased levels of child anxiety and reduced intrinsic motivation to learn (33). Inappropriate educational methods further exacerbate these issues. Some parents adopt punitive or coercive strategies instead of developmentally appropriate guidance. As one child reported, “If I score low, my mom just yells or compares me to others.” These methods often include scolding, comparison, or conditional affection, which risk damaging children’s self-esteem and internal motivation. Research has found that authoritarian parenting is linked to poorer academic adjustment and lower emotional well-being in children (34).

### 4.2 Causes of the inadequate family care for children

Inadequate family care for children is shaped by a complex interplay of internal and external factors, many of which are closely tied to China’s ongoing social transformation. Internally, instability within family caregiving structures often disrupts continuity of care. This is particularly evident in single-parent families and families in which grandparents serve as primary caregivers. In these contexts, caregiving responsibilities are frequently redistributed or inconsistently applied, weakening the emotional and developmental support available to children in need. For example, a grandmother caring for her grandson in a rural area noted, “I cook and keep him safe, but I do not know how to help with his feelings or schoolwork.” Such limitations are not due to unwillingness but reflect generational and educational gaps in caregiving capacity (35). Additionally, a lack of scientific knowledge about child development hinders many parents from providing appropriate care (36). Some parents admitted that they “just follow how their parents raised them,” despite recognizing that today’s children face different challenges than they did. One father commented during an interview: “I just raise him the way my parents raised me. I went through it like that, so I think he can too.” In families with young parents, especially those in urban environments, this knowledge gap often manifests as ineffective emotional regulation or educational strategies. One urban mother noted: “I tell my daughter to just calm down, but I do not know how to teach her to understand her emotions instead.” Furthermore, internal conflicts between work and caregiving roles are common. Parents frequently expressed guilt and helplessness about not having enough time or energy for their children. One father explained, “I work overtime six days a week—when I get home, I just want to sleep, not argue over homework.” Even parents who are motivated to be involved may lack the practical skills or emotional tools needed to provide consistent and high-quality care.

External factors further compound these challenges, particularly those tied to China’s rapid urbanization and labor migration. Many families in rural areas rely on grandparents to care for children while parents migrate to cities for work (37). These left-behind children often lack daily emotional support and direct guidance (38). One child remarked, “I talk to my mom on video sometimes, but it’s not the same. I miss her when I’m sad.” In urban areas, dual-income families face different but equally demanding pressures. Limited access to affordable childcare, rigid work schedules, and the absence of after-school support leave many parents struggling to balance career and caregiving. A mother shared, “If there were after-school programs, I would not feel so guilty about working late.” Social and cultural expectations also contribute to caregiving stress. The exam-oriented education system places disproportionate emphasis on academic performance, leading parents to focus heavily on grades at the expense of emotional development (39). As one caregiver put it, “If he does not get good grades, he will not have a future—that’s the reality.” Traditional gender roles continue to influence caregiving dynamics, with mothers often bearing the primary caregiving burden while fathers remain less involved (40). A mother remarked, “My husband works late and says he’s too tired, so most things fall to me.” These role expectations intensify stress and limit shared caregiving. Besides, we observed that younger urban parents reported more anxiety about screen time and academic competition, while rural caregivers—especially grandparents—focused more on safety and nutrition. One urban parent stated, “I’m more worried about him being addicted to the phone than eating candy.” In contrast, a rural grandparent noted, “As long as he eats well and does not get hurt, It’s enough.” These differences suggest that inadequate caregiving is not a monolithic issue but varies by region, age, and caregiver role (see Supplementary Table A1). In conclusion, the causes of inadequate family care are deeply rooted in both household-level limitations and broader structural pressures.

To address these caregiving challenges, several practical policy measures should be considered. These include promoting more flexible working arrangements for parents, expanding access to affordable after-school care and community-based childcare services, and offering accessible parenting education programs focused on emotional communication, screen time management, and modern caregiving strategies. Tailored support for grandparent caregivers, particularly in rural and migrant-sending areas, is also essential. Such efforts would help align caregiving resources with the evolving needs of families and contribute to improving the quality and consistency of family care across diverse social contexts in China.

### 4.3 The consequence of the inadequate family care for children

Inadequate family care has significant negative impacts on children’s growth and development, affecting key domains such as parent–child relationships, social skills, behavior, emotional regulation, and physical health (41). Research by Wu et al. reveals a strong correlation between lower levels of parental involvement and poorer quality of parent–child attachment, which in turn increases the risk of psychological and behavioral problems (42). When parents fail to provide adequate time, energy, and emotional support, the frequency and quality of interactions diminish, often leading children to feel neglected or misunderstood. One child shared, “My dad is always busy. When I talk to him, he just says ‘later’ but later never comes.” Another expressed, “Sometimes I do not tell them things because they are always on their phones or angry.” These sentiments reflect how emotional unavailability can weaken children’s sense of trust and security, resulting in reduced communication, increased conflict, and gradual erosion of the parent–child bond (43). The family serves as the first and most influential environment for a child’s socialization, where social norms, emotional expression, and interpersonal behavior are first learned (44). A lack of adequate emotional and behavioral guidance from caregivers can hinder a child’s ability to build peer relationships, regulate emotions, and integrate into social settings (45). A child also noted, “I get angry easily because they cannot do anything about me.” These deficiencies may present as social withdrawal, difficulty forming friendships, or limited social adaptability. Studies have shown that consistent parental support and involvement not only enhance children’s social development but also improve the quality of peer interactions and promote emotional cooperation (46).

Moreover, insufficient emotional care heightens the risk of emotional and mental health issues such as anxiety, depression, and loneliness. In emotionally unresponsive or psychologically neglectful environments, children may struggle with emotional regulation, often exhibiting instability, low self-worth, or suppressed emotions (47). One father remarked, “I did not realize my son was feeling so lonely until his teacher called us.” These narratives reflect a lack of emotional validation and support, which over time erode psychological resilience. Behaviorally, the lack of stable caregiving and clear boundaries may impair children’s understanding of acceptable conduct, increasing the likelihood of disciplinary issues or antisocial behavior. Children who lack consistent emotional affirmation may express distress through aggression, defiance, self-harm, or other disruptive actions (48). A parent shared, “He throws tantrums and breaks things—I think it’s his way of telling us he needs more attention.”

In parallel, inadequate family care also jeopardizes children’s physical health. Without regular health monitoring, nutritional guidance, and structured routines, children are more prone to malnutrition, stunted growth, and other preventable health issues (49). One grandparent explained, “We only found out our grandchild was anemic because the school health check told us.” A study on the prevalence of obesity among Chinese children and adolescents reported that the rates of overweight and obesity reached 27.2 and 29.6%, respectively, reflecting the consequences of poor dietary habits and insufficient parental oversight (50). Additionally, irregular daily schedules, poor sleep quality, and missed health check-ups are commonly linked to limited family involvement, further compromising children’s overall well-being. One mother shared: “I know my son stays up too late and skips breakfast, but I leave early for work and just hope he manages on his own.” Collectively, these emotional, behavioral, social, and physical consequences underscore the urgent need for comprehensive family support systems to foster healthy, balanced development in children.

## 5 Conclusion

Using grounded theory, this study offers an in-depth analysis of inadequate family care for children, establishing a conceptual framework that elucidates its core attributes, antecedents, and impacts on children’s development. The findings revealed that inadequate family care spans four primary dimensions: daily living care, emotional and psychological support, safety care, and educational guidance. Through a detailed analysis, both internal and external factors were identified as antecedents, collectively contributing to deficiencies in one or more of these caregiving dimensions. This study emphasizes the extensive repercussions of inadequate care on children’s physical and mental development, underscoring the importance of addressing this issue. The findings suggest that raising parental awareness of caregiving responsibilities, enhancing social support systems, and strengthening policy guidance are essential steps to ensure adequate care for children. Such measures could help alleviate the adverse effects of inadequate family care, ultimately promoting comprehensive physical and mental well-being among children.

## Data Availability

The raw data supporting the conclusions of this article will be made available by the authors, without undue reservation.
