# Supplementary material for: Uncovering the gaps: a grounded theory approach to conceptualizing inadequate child family caregiving in China
Source: Front Public Health. 2025 Aug 6;13:1539227. doi: 10.3389/fpubh.2025.1539227 (PMC12364826; doi:10.3389/fpubh.2025.1539227)
Supplement: Supplementary file 1 [file Table_1.docx]

**Supply Table A1 The frequency of main category and the corresponding subcategory**

| **Main Category** | **Subcategory** | **Initial Category** | **No. of mentions by Children** | **No. of mentions by caregivers** | **Residence** | | **Family Type** | | | | **Age of Caregiver** | | **Caregiver Type** | |
| --- | --- | --- | --- | --- | --- | --- | --- | --- | --- | --- | --- | --- | --- | --- |
|  |  |  |  |  | **Urban** | **Rural** | Core family | Left-behind family | Single-parent family | Immigrate family | **<40** | **≥40** | **Parents** | **Grandparents** |
| Inadequate in daily living care | Inadequate Nutritional Care | Nutritional Imbalance in Children's Food | 18 | 22 | 71.4% | 64.3% | 46.1% | 66.7% | 66.7% | 50.0% | 61.5% | 60.0% | 54.5% | 66.7% |
|  |  | Excessive Intake of Unhealthy Foods by Children | 15 | 20 | 85.7% | 57.1% | 61.5% | 83.3% | 66.7% | 50.0% | 69.2% | 66.7% | 63.6% | 66.7% |
|  |  | Poor Eating Habits in Children | 14 | 16 | 71.4% | 57.1% | 53.8% | 66.7% | 66.7 % | 50.0% | 76.9% | 66.7% | 59.1% | 66.7% |
|  | Insufficient Rest and Physical Activity Care | Insufficient Sleep and Rest Time for Children | 12 | 19 | 64.3% | 50.0% | 46.1% | 66.7% | 50.0% | 33.3% | 69.2% | 66.7% | 54.5% | 66.7% |
|  |  | Irregular Sleep Patterns in Children | 8 | 18 | 64.3% | 57.1% | 38.4% | 66.7% | 50.0% | 33.3% | 76.9% | 60.0% | 59.1% | 66.7% |
|  |  | Insufficient Physical Activity in Children | 10 | 17 | 50% | 46.1% | 38.4% | 50.0% | 33.3% | 50.0% | 61.5% | 73.3% | 77.3% | 66.7% |
|  | Lack of Health Monitoring and Medical Care | Lack of Regular Health Monitoring for Children | 9 | 13 | 85.7% | 57.1% | 46.1 % | 100% | 83.3% | 66.7% | 61.5% | 66.7% | 68.2 % | 83.3% |
|  |  | Improper Handling of Children's Health Symptoms | 10 | 11 | 92.8% | 57.1% | 38.4% | 66.7% | 50.0% | 66.7% | 53.8% | 60.0% | 59.1% | 83.3% |
|  | Inadequate Hygiene and Cleanliness Care | Poor Personal Hygiene Habits in Children | 9 | 13 | 71.4% | 42.8% | 30.7% | 66.7% | 50.0% | 33.3% | 46.1% | 46.6% | 59.1% | 66.7% |
|  |  | Untimely Household Cleanliness | 8 | 11 | 64.3% | 42.8% | 30.7% | 66.7% | 50.0% | 50.0% | 30.7% | 33.3% | 59.1% | 66.7% |
| Inadequate in emotional and psychological care | Lack of Companionship | Limited Parent-Child Time | 21 | 17 | 64.3% | 85.7% | 53.8% | 83.3% | 66.7% | 66.7% | 46.1% | 46.6% | 63.6% | 66.7% |
|  |  | Lack of High-Quality Parent-Child Interaction | 19 | 16 | 64.3% | 71.4% | 76.9 % | 83.3% | 83.3% | 66.7 % | 76.9% | 73.3% | 77.3% | 66.7% |
|  | Insufficient Affection | Limited Parental Emotional Expression | 20 | 15 | 71.4% | 92.8% | 69.2 % | 83.3% | 83.3% | 66.7 % | 84.6% | 73.3% | 81.8% | 66.7% |
|  |  | Neglect of Children’s Psychological and Emotional Needs | 23 | 14 | 71.4% | 85.7% | 61.5% | 83.3% | 83.3% | 66.7 % | 84.6% | 66.7% | 81.8% | 66.7% |
|  | Lack of Understanding | Ignoring Children’s Expressions | 21 | 17 | 85.7% | 92.8% | 76.9% | 83.3% | 83.3% | 83.3% | 84.6% | 73.3% | 86.3% | 83.3% |
|  |  | Lack of Understanding of Children's Developmental Processes | 14 | 12 | 64.3% | 85.7% | 76.9% | 100% | 83.3% | 66.7% | 84.6% | 66.7% | 86.3% | 66.7% |
|  | Insufficient Support | Insufficient Psychological Support | 18 | 19 | 71.4% | 85.7% | 69.2% | 83.3% | 100% | 83.3% | 84.6% | 73.3% | 81.8% | 66.7% |
|  |  | Insufficient Emotional Support | 20 | 15 | 64.3% | 71.4% | 76.9% | 83.3% | 83.3% | 83.3% | 84.6% | 80.0% | 90.9% | 83.3% |
| Inadequate in safety care | — | Inadequate Safety Supervision | 12 | 16 | 64.3% | 57.1% | 53.8% | 100% | 83.3% | 83.3% | 46.1% | 33.3% | 45.5% | 33.3% |
|  | — | Limited Content or Methods in Safety Education | 10 | 13 | 64.3% | 64.3% | 53.8% | 83.3% | 100% | 66.7% | 53.8% | 53.3% | 59.1% | 50.0% |
| Inadequate in educational care | — | Lack of Behavior Habit Development Education | 11 | 9 | 42.8% | 64.3% | 30.7% | 83.3% | 66.7% | 50.0% | 84.6% | 73.3% | 81.8% | 66.7% |
|  | — | Misplaced Educational Focus | 18 | 12 | 57.1% | 35.7% | 53.8% | 83.3% | 83.3% | 66.7% | 84.6% | 73.3% | 86.3% | 66.7% |
|  | — | Inappropriate Parenting Methods | 17 | 10 | 71.4% | 64.3% | 46.1% | 83.3% | 100% | 50.0% | 84.6% | 66.7% | 59.1% | 83.3% |
| Internal Factors Contributing to inadequate family care for children | — | Unstable Family Care Structure | 13 | 12 | 35.7% | 92.8% | 23.1% | 100% | 100% | 33.3% | 38.4% | 40.0% | 45.5% | 66.7% |
|  | Caregiver-Related Factors | Insufficient Parenting Knowledge | - | 23 | 57.1% | 92.8% | 38.4% | 83.3% | 83.3% | 50.0% | 53.8% | 40.0% | 45.5% | 66.7% |
|  |  | Lack of Motivation in Parenting | - | 20 | 64.3% | 57.1% | 46.1% | 83.3% | 100% | 50.0% | 76.9% | 73.3% | 81.8% | 50.0% |
|  |  | Limited Parenting Skills/Capabilities | - | 22 | 57.1% | 85.7% | 46.1% | 83.3% | 66.7% | 50.0% | 53.8% | 66.7% | 59.1% | 66.7% |
| External Factors Contributing to inadequate family care for children | — | Insufficient Emotional Support within the Family | - | 17 | 57.1% | 64.3% | 38.4% | 83.3% | 100% | 33.3% | 53.8% | 53.3% | 59.1% | 50.0% |
|  | Social Factors | Weak Social Support System | - | 20 | 57.1% | 71.4% | 30.7% | 83.3% | 83.3% | 33.3% | 76.9% | 60.0% | 59.1% | 66.7% |
|  |  | Societal and Cultural Pressure | - | 17 | 64.3% | 64.3% | 46.1% | 66.7% | 66.7% | 66.7% | 76.9% | 60.0% | 81.8% | 50.0% |
| Adverse Outcomes of Inadequate Family Care for children | — | Decline in Parent-Child Relationship Quality | 18 | 20 | 57.1% | 64.3% | 30.7% | 66.7% | 83.3% | 33.3% | 53.8% | 53.3% | 54.5% | 50.0% |
|  | Physical and Mental Developmental Issues in Children | Limited Social Development in Children | 10 | 18 | 57.1% | 57.1% | 46.1% | 66.7% | 83.3% | 66.7% | 46.1% | 53.3% | 54.5% | 50.0% |
|  |  | Increase in Behavioral Problems in Children | 7 | 15 | 71.4% | 64.3% | 38.4% | 83.3% | 100% | 66.7% | 53.8% | 73.3% | 68.2% | 66.7% |
|  |  | Emotional Imbalance in Children | 12 | 16 | 71.4% | 64.3% | 46.1% | 66.7% | 83.3% | 50.0% | 53.8% | 60.0% | 72.7% | 66.7% |
|  |  | Health Issues in Children | 5 | 13 | 42.8% | 42.8% | 23.1% | 66.7% | 50.0% | 33.3% | 46.1% | 40.0% | 36.4% | 50.0% |
